# The role of FAPI PET/CT in patients with lymphoma: a systematic review

**DOI:** 10.3389/fnume.2025.1589903

**Published:** 2025-05-09

**Authors:** Natale Quartuccio, Stefania Nicolosi, Sabina Pulizzi, Dante D’Oppido, Salvatore Ialuna

**Affiliations:** Nuclear Medicine Unit, Ospedali Riuniti Villa Sofia-Cervello, Palermo, Italy

**Keywords:** positron emission tomography, FDG, FAPI, lymphoma, NHL, DLBCL, systematic review

## Abstract

**Introduction:**

Fluorodeoxyglucose (FDG) PET/CT is typically the reference imaging method for assessing and tracking lymphomas. However, fibroblast activation protein inhibitor (FAPI) PET is being explored as a potentially useful option, especially when Fluorodeoxyglucose (FDG) scans do not show clear results.

**Methods:**

For this systematic review, two researchers searched PubMed/MEDLINE and Cochrane CENTRAL for studies on FAPI PET/CT in lymphoma patients.

**Results:**

The literature search initially retrieved 249 articles. After removing duplicates and screening titles and abstracts, and full text, there was a final selection of 15 articles (3 original studies and 12 case reports), encompassing a total of 270 patients. The three original studies were judged to have a low risk of bias according to the QUADAS-2 criteria. The systematic review reveals that FAPI PET/CT exhibits lower diagnostic sensitivity than [^18^F]FDG PET/CT in lymphomas characterized by low FAP expression. Nevertheless, FAPI PET/CT retains potential as a complementary imaging modality.

**Discussion:**

[^18^F]FDG PET/CT remains the gold standard in lymphoma imaging, but FAPI PET/CT can potentially provide supplementary information regarding the molecular characteristics of lymphomas. FAPI PET/CT may have prognostic and therapeutic implications. In particular, it could help identify lymphoma subgroups with distinct stromal environments, potentially serving as a prognostic biomarker. Further large-scale prospective studies are warranted to validate its role in lymphoma management.

## 1 Introduction

Lymphoma, a type of cancer that originates in the lymphatic system, encompasses various subtypes, including Non-Hodgkin's lymphoma (NHL), the most prevalent globally. In 2020, NHL alone accounted for over half a million new diagnoses and a quarter of a million deaths worldwide. While generally, more common in developed countries, certain regions, such as North Africa, experienced disproportionately high mortality rates. Moreover, NHL cases are rising significantly in countries like Australia and New Zealand. A particularly concerning trend is the substantial rise in both incidence and mortality among elderly populations over the past few decades. Due to changing demographics, projections indicate that total lymphoma cases, largely driven by NHL, will likely reach nearly 800,000 by 2040 (1–3). Hodgkin lymphoma (HL) is a malignancy of B cells, distinguished by the presence of Reed–Sternberg cells within an inflammatory microenvironment. It represents approximately 10% of all lymphoma cases and has a global incidence of about 2–3 per 100,000 people annually. HL follows a bimodal age distribution, with incidence peaks in young adults (ages 15–35) and again in individuals over 55, with a slightly higher prevalence in males than females (4). Positron emission tomography/computed tomography (PET/CT), specifically when using fluorine-18-labeled fluorodeoxyglucose ([^18^F]FDG) as radiotracer, is a crucial imaging modality for visualizing metabolic activity, particularly in rapidly dividing cancer cells. This imaging technique is valuable for identifying residual or recurrent disease, where [^18^F]FDG PET/CT can detect areas of hypermetabolism indicative of residual tumor cells after primary treatment or disease recurrence, often with higher sensitivity than conventional imaging techniques (5, 6). Furthermore, [^18^F]FDG imaging allows for the quantification of metabolic changes induced by therapy, providing an early indicator of treatment effectiveness and enabling timely adjustments to the therapeutic strategy. Finally, information obtained through [^18^F]FDG PET/CT contributes to better prognostic stratification of patients and supports the clinical decision-making process, guiding the choice of personalized therapies and the most appropriate follow-up (7, 8).

The introduction of fibroblast activation protein inhibitor (FAPI) as a PET radiotracer represented a significant advancement in cancer imaging. FAPI demonstrates superior tumor uptake and contrast compared to the conventional [^18^F]FDG in various malignancies, including, but not limited to, gastric, colorectal, and breast cancers (9, 10). This enhanced detection rate is attributed to FAPI's selective targeting of fibroblast activation protein (FAP), which is overexpressed in cancer-associated fibroblasts within the tumor microenvironment, and is considered a marker for pro-tumorigenic stroma (11). A recent systematic review confirmed the higher sensitivity and specificity of FAPI PET/CT in detecting and staging several cancer types. However, the potential utility of FAPI in lymphoma imaging remains relatively unexplored (9). While preliminary studies suggest promising results, further research is warranted to evaluate the diagnostic accuracy and clinical implications of FAPI PET/CT in lymphoma, particularly in characterizing lymphoma subtypes (12, 13).

The aim of this study is to provide a systematic review of the literature on the studies using FAPI PET/CT in patients with lymphoma.

## 2 Methods

This systematic review followed the Preferred Reporting Items for Systematic Reviews and Meta-Analyses (PRISMA) 2020 guidelines (14). A detailed protocol was estabilished beforehand, outlining the research question, search strategy, criteria for including studies, quality assessment methods, data extraction process, and planned statistical analyses. The protocol of the systematic review was not registered in any public register.

### 2.1 Literature search

Two researchers searched the PubMed/MEDLINE and Cochrane CENTRAL databases for studies using [^18^F]FAPI PET/CT in lymphoma patients. The search was conducted on January 18, 2025, at 12 pm Bethesda time, with no restrictions on language or publication date.

The search string for the literature search in PubMed/MEDLINE was: {[“68Ga-FAPI” (Supplementary Concept)] OR FAPI} AND (“Positron Emission Tomography Computed Tomography”[Mesh] OR PET) AND (“Lymphoma”[Mesh] OR lymp*).

The string used for the search in CENTRAL was “FAPI AND lymphoma”.

The literature search was updated until 15 February 2024, Bethesda, time: 12 pm, for both databases.

### 2.2 Study selection

All identified references were exported to Endnote v. X7.5. Due to the limited data available in literature and specific scope of the present review topic, even case reports were included in the study selection, although they are not typically examined in systematic reviews. A researcher screened titles and abstracts to remove duplicates, irrelevant articles, reviews and meta-analyses. Full texts of the remaining articles were retrieved and assessed for eligibility based on the execution of a FAPI PET/CT in patients with lymphoma. Original studies were included if they involved a (1) cohort or subset of lymphoma patients undergoing FAPI PET/CT, and (2) the patients had no history of other concomitant or previous malignancies. If a full text was unavailable, the corresponding author was contacted. Finally, the reference lists of the included articles were checked for additional relevant studies.

### 2.3 Data extraction

Two researchers independently extracted data from all included studies in duplicate. Any discrepancies were resolved through discussion and consensus, with the option to consult a third researcher. Bibliographic and technical information from the articles was then compiled into a descriptive table.

### 2.4 Methodological quality assessment

One investigator evaluated the methodological quality of the included original studies using the QUADAS-2 tool (version 2) (15, 16). This tool assesses four domains: patient selection, index test, reference standard, and flow and timing. Risks of bias and concerns about applicability were categorized as low, high, or unclear.

## 3 Results

### 3.1 Literature search and eligibility assessment

**Figure 1:**
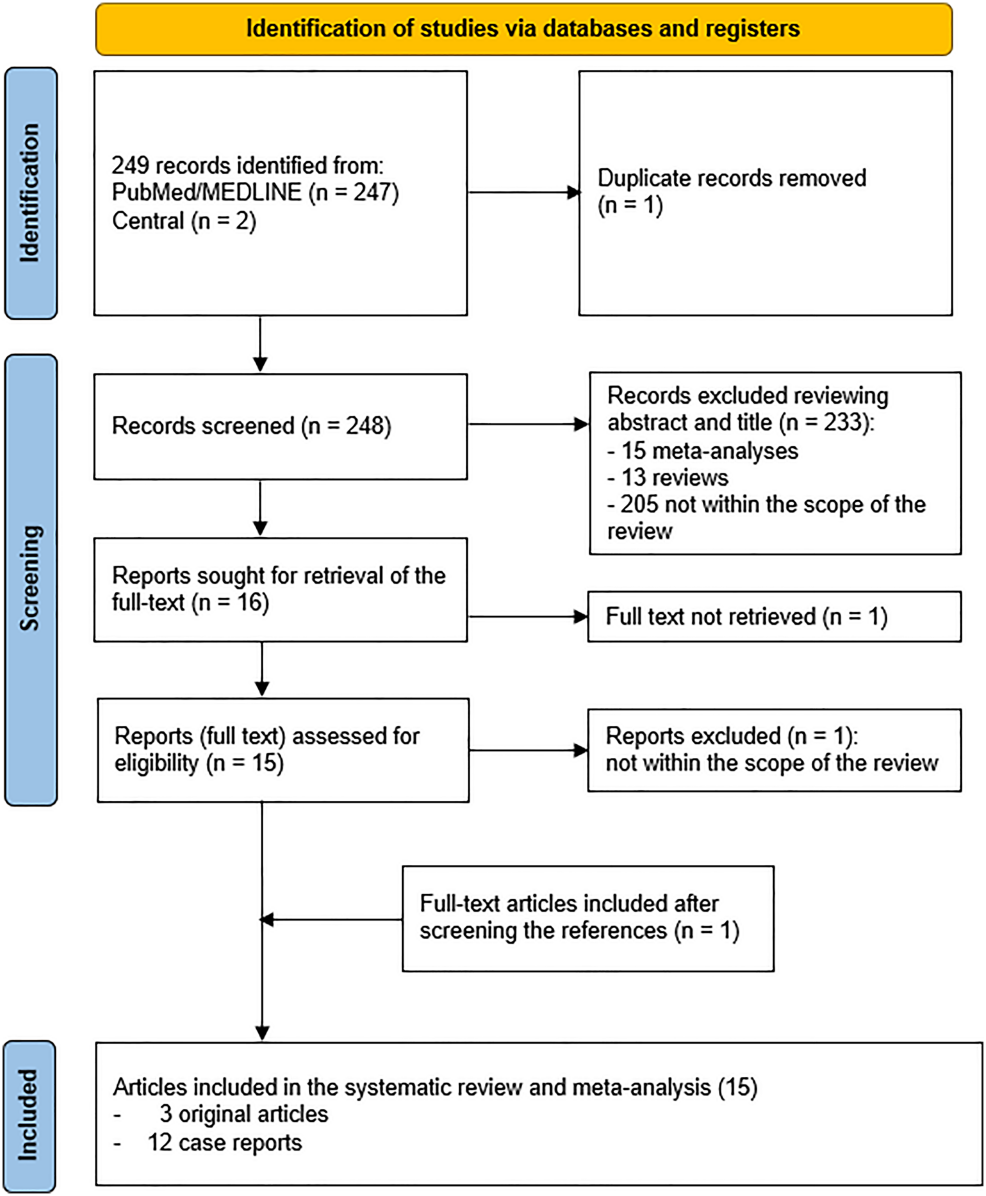
Flow-chart of the literature search.

The comprehensive computer literature search revealed 249 articles (Figure 1). After importing the articles in the reference manager, one article was removed because it was a duplicate, leading to 248 entries. Reviewing titles and abstracts, 233/248 entries were excluded because they did not meet the inclusion criteria of the systematic review (15 meta-analyses, 13 reviews and 205 articles not within the scope of the review). The full text of the remaining 16 entries was sought for retrieval to check the inclusion criteria. Among the 16 articles, 1 full text (17) could not be downloaded and was not provided by the corresponding author despite formal email request. One out of 15 full texts (18) was not within the field of interest of the systematic review, since reported a case of thymic squamous cell carcinoma mimicking lymphoma on [^68^Ga]FAPI PET/CT. One additional record was retrieved (19) and included in the systematic review after crosschecking the references, leading to a final selection of 15 articles (3 original articles and 12 case reports). The main characteristics of the 15 articles with a total number of 270 patients included in the systematic review are presented in Table 1.

**Table 1:** Characteristics of the studies included in the systematic review.

Authors	Year	Country	Journal	Number of patients	Sex	Age	Type of lymphoma	FAPI tracer	SUVmax FAPI	SUVmax FDG
Wang et al.	2025	China	Clin Nucl Med	1	F	56	MALT: primary pulmonary MALT	68Ga-FAPI	8.1	3.7
Hirmas et al.	2024	Germany	J Nucl Med	10	NR	NR	NR	68Ga-FAPI	NR	NR
Ran et al.	2024	China	Clin Nucl Med	1	M	22	T-cell lymphoma: primary peripheral T-cell lymphoma of the skeletal muscles with brain involvement	18 F-FAPI-42	Muscles: 4.9 Brain lesion: 0.9	Muscles: 21.3 Brain lesion: 8.9
Liu et al.	2024	China	Clin Nucl Med	1	F	44	T-cell lymphoma: Mycosis fungoides-type cutaneous T-cell lymphoma	18 F-FAPI-42	Skin: 11.3 Inguinal lymph nodes: 3.8	Skin: 7.7 Inguinal lymph nodes: 5.2
Chen et al.	2023	China	J Nucl Med	186	91 M; 95 F	52 (median)	HL 24 (12.9%) Non-Hodgkin lymphoma 162 (87.1%) DLBCL 80 (43.0%) Primary mediastinal large B-cell lymphoma 4 (2.2%) Mantle cell lymphoma 5 (2.7%) Burkitt lymphoma 2 (1.1%) Lymphoblastic leukemia/lymphoma 6 (3.2%) PTCL 18 (9.7%) Extranodal NK/T-cell lymphoma 6 (3.2%) FL 31 (16.7%) MALT 8 (4.3%) Chronic lymphocytic leukemia/small lymphocytic lymphoma	68Ga-FAPI	Nodal lesions: HL: 8.6 NHL 7.3 Extranodal lesions: HL: 7.9 NHL: 7.1	Nodal lesions: HL: 11.6 NHL: 10.5 Extranodal lesions HL: 10.1 NHL: 11
Lu et al.	2023	China	Clin Nucl Med	1	M	73	DLBLC	18 F-FAPI	negative	18.9
Wu et al.	2022	China	Diagnostics (Basel)	1	M	82	T-Cell Lymphoma: Angioimmunoblastic T-Cell Lymphoma	68Ga-FAPI	1.6	7.6
Jin et al.	2022	China	J Nucl Med	73 (11 HL, 63 NHL)	36 F; 37 M	51.6 ± 14.2	HL, NHL	68Ga-FAPI	9.46	N/A
Pang et al.	2022	China	Clin Nucl Med	1	M	76	MALT: primary hepatic extranodal marginal zone lymphoma of mucosa-associated lymphoid tissue	68Ga-FAPI	1.56 higher TBR than 18F-FDG (1.6 vs. 0.7).	2.8
Kou et al.	2022	China	Clin Nucl Med	1	M		MALT: mucosa-associated lymphoid tissue lymphoma with secondary liver involvement.	Al^18^F-NOTA-FAPI-04	Hepatic lesion: 4.9	Hepatic lesion: no abnormal uptake
Chen et al.	2022	China	Clin Nucl Med	1	M	67	Follicular lymphoma	68Ga-FAPI-04	Stomach: 15.1 Lymph nodes: 5.8	Stomach: 6 Lymph nodes: 10
Yang et al.	2022	China	J Nucl Cardiol	1	F	32	DLBLC: mediastinal lymphoma	68Ga-FAPI	13	N/A
Yang et al.	2021	China	Endocrine	1	F	60	DLBLC: thyroid lymphoma	68Ga-FAPI	8.6	N/A
Zhang	2021	China	Clin Nucl Med	1	F	67	DLBLC: primary central nervous system lymphoma	68Ga-FAPI	CNS lesion: 3.1	CNS lesion: 9.6
Wang	2020	China	Clin Nucl Med	1	F	55	DLBLC: primary gastric lymphoma	68Ga-NOTA-FAPI-04	Stomach: 5 Lymph nodes: 1.4	Stomach: 2.9 Lymph nodes: 2

NR, not reported; MALT, mucosa-associated lymphoid tissue; DLBLC, diffuse large B-cell lymphoma.

### 3.2 Methodological quality of included studies

The three original studies included in the systematic review were assessed as having a low risk of bias according to the QUADAS-2 criteria (Table 2; Supplementary Table S1).

**Table 2:** Representation evaluating the quality of the studies included in the systematic review based on the four domains of the risk of bias (patient selection, index test, reference standard, flow and timing) and the three domains of the applicability concerns (patient selection, index test, reference standard).

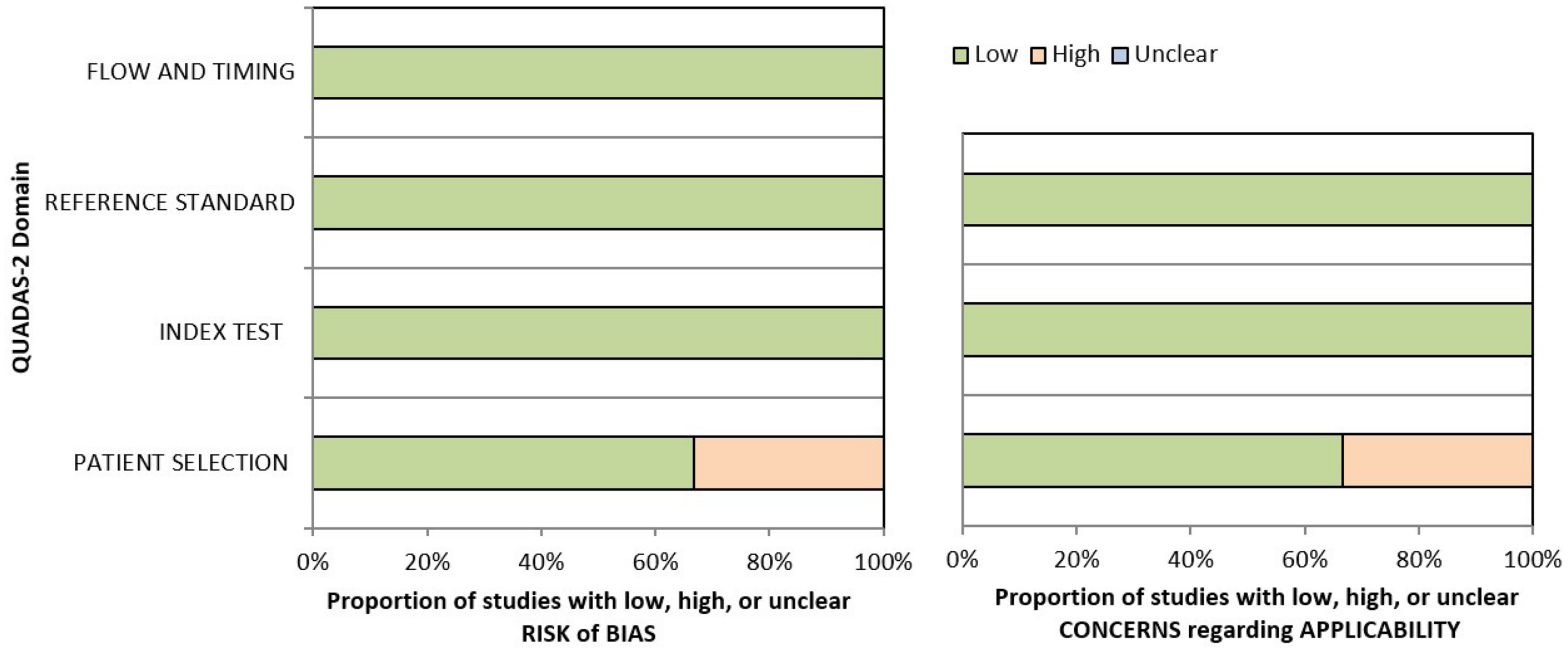

### 3.3 Systematic review

#### 3.3.1 Original studies

Only three original articles including a cohort or a subgroup of patients with lymphoma have been published in literature so far (12, 13, 19). In the study by Jin et al. [^68^Ga]FAPI PET/CT scans were performed on 73 lymphoma patients (11 with Hodgkin lymphoma and 62 with non-Hodgkin lymphoma). FAP levels were measured both in the scans and in tissue samples from 22 patients. Elevated FAP uptake was notably higher in Hodgkin lymphoma lesions, which correlated with intense FAP immunostaining (score, 31). In non-Hodgkin lymphoma, a positive association was observed between clinical classification and [^68^Ga]FAPI uptake. Aggressive NHL lesions, with moderate to strong FAP immunostaining (scores between 21 and 31), showed higher levels of [^68^Ga]FAPI uptake. In contrast, indolent NHL lesions had weaker FAP staining and displayed mild to moderate FAPI uptake. No significant correlation was found between the sum of the product of diameters and the corresponding SUVmax (*P* = 0.424). Tumor-to-liver ratios were 6.26 ± 4.17 in indolent NHL cases and over 9 in other lymphoma subtypes (13). In conclusion, [^68^Ga]FAPI PET/CT can effectively detect FAP in lymphoma lesions, potentially serving as an additional tool to characterize lymphomas.

In a small subgroup of mixed lymphoma patients (*n* = 10), [^68^Ga]FAPI PET was found to be less effective than [^18^F]FDG PET. Specifically, when looking at individual patients, [^68^Ga]FAPI PET was less accurate, less able to correctly identify those with lymphoma, and less reliable in ruling out lymphoma compared to [^18^F]FDG PET. Similarly, when examining specific areas of the body, [^68^Ga]FAPI PET again showed lower accuracy, reduced ability to detect lymphoma, and decreased reliability in excluding lymphoma compared to [^18^F]FDG PET. Essentially, [^18^F]FDG PET performed better in all measured categories (19).

Another pivotal study compared [^68^Ga]FAPI and [^18^F]FDG PET/CT in lymphoma, examining FAP and glycolytic marker uptake. 186 patients underwent both scans, with immunohistochemistry assessing FAP, hexokinase 2, and GLUT1. Statistical analyses included t-tests, Wilcoxon, and Spearman correlation. [^18^F]FDG PET had higher staging accuracy (98.4%) than [^68^Ga]FAPI PET (86.0%), detecting more nodal (4,624 vs. 2,196) and extranodal (1,304 vs. 845) lesions. 52 lesions were [^68^Ga]FAPI positive and [^18^F]FDG negative; 2,939 were vice versa. No significant SUVmax or target-to-liver ratio differences were found between tracers in some lymphoma subtypes. GLUT1 and hexokinase 2 were overexpressed in lymphoma cells and the microenvironment, while FAP was stromal. FAP correlated with [^68^Ga]FAPI SUVmax (r = 0.622, *P* = 0.001), GLUT1 with [^18^F]FDG SUVmax (r = 0.835, *P* < 0.001) (12).

#### 3.3.2 Case reports: MALT

In the case reported by Wang et al., an elderly woman with cough and sputum had a chest CT revealing a solid lesion in the left upper lobe. After 2 weeks of antibiotics and no change on a repeat CT, she underwent both [^18^F]FDG and [^68^Ga]DOTA-FAPI-04 PET/CT scans. The [^18^F]FDG PET/CT showed a 1.6 × 1.4 cm lesion with mild uptake (SUVmax 3.71), while the [^68^Ga]FAPI PET/CT demonstrated higher uptake (SUVmax 8.10), indicating FAP expression. No lymph node or other metastases were seen. Surgical resection and subsequent histopathology confirmed the lesion as pulmonary marginal zone lymphoma of mucosa-associated lymphoid tissue (MALT lymphoma) (20). In keeping with this cases, Pang and colleagues demonstrated the utility of ^68^Ga-FAPI. In their case, a 76-year-old male was diagnosed with a rare primary hepatic extranodal marginal zone lymphoma of MALT, confirmed through pathological examination. [^18^F]FDG PET/CT was performed as part of the initial diagnostic workup. Subsequent [^68^Ga]FAPIPET/CT revealed a significantly higher tumor-to-background ratio in the hepatic lesion compared to the [^18^F]FDG scan. This enhanced uptake was attributed to minimal [^68^Ga]FAPIuptake in the surrounding normal liver tissue (21). Similarly, a man was diagnosed with a MALToma with secondary liver involvement. The liver lesion did not show FDG PET/CT uptake, but only abnormal FAPI accumulation, suggesting the usefulness of FAPI PET/CT in detecting hepatic mucosa-associated lymphoid tissue lymphoma (22). These cases suggest that Ga-FAPI PET/CT may be an alternative imaging method to characterize low-grade lymphomas.

#### 3.3.2 Case reports: follicular lymphoma

Other authors demonstrated the utility of FAP in follicular lymphoma. In a case report, a 67-year-old man was found to have both follicular lymphoma and stomach cancer. Initial PET scans using [^18^F]FDG showed high activity in his lymph nodes and a thickened area in his stomach. However, when scanned with [^68^Ga]FAPI-04, the stomach lesion showed strong uptake while the lymph nodes showed only slight uptake. After treatment, his lymph nodes were completely clear, but the stomach lesion showed even more tracer uptake than before. Although doctors initially thought the lymphoma might have changed to a more aggressive type, the scans accurately identified and differentiated the two distinct cancers (23).

#### 3.3.3 Case reports: T-cell lymphoma

A group of authors describe an 82-year-old man, previously treated for colon cancer, who was found to have enlarged lymph nodes and a lung tumor. A first FDG-PET/CT scan showed very high uptake in both the lymph nodes and the lung mass, suggesting the colon cancer had spread. However, the pattern of lymph node enlargement made doctors suspect another condition like lymphoma or inflammation. Then, a FAPI-PET/CT scan revealed very low uptake in the lymph nodes suggesting that the lymph nodes were not related to the lung mass. A biopsy of a neck lymph node confirmed the patient had angioimmunoblastic T-cell lymphoma. Additionally, the FAPI-PET scan revealed a highly active area in the prostate, which, combined with a high PSA level, pointed to prostate cancer (24). Another group of authors reported a 22-year-old man presented with increasing limb weakness and numbness. Brain and neck MRIs showed enhancing lesions in multiple brain regions, suggestive of metastases. An FDG PET/MRI revealed numerous FDG-avid areas in his skeletal muscles and brain. The muscle tumors displayed intense FDG uptake, with a SUVmax of 21.3, and mild to moderate FAPI uptake, with a SUVmax of 4.9, indicating FDG's better ability to visualize muscle tumors. FDG PET highlighted intense uptake in muscles like the trapezius, obliquus capitis inferior, and pectoralis major. A FAPI PET scan, performed two days later, showed FAPI-avid areas with lower uptake, ranging from a SUVmax of 0.9 to 4.9. Notably, FAPI PET was superior for visualizing brain lesions due to its low background activity. A biopsy confirmed peripheral T-cell lymphoma, and chemotherapy led to a complete response (25). These cases demonstrate that FAPI-PET scans may be useful in differentiating solid tumors from hematologic malignancies by revealing differences in the extent of peritumoral fibrosis. Furthermore, they illustrate the complementary roles of FDG and FAPI PET in T-cell lymphoma, highlighting FDG's efficacy in muscle tumor detection and FAPI's advantage in brain lesion delineation.

#### 3.3.4 Case reports: DLBCL

Wang et al. reported a 55-year-old woman with fatigue, weight loss, and upper abdominal pain and normal tumor marker blood tests. A CT scan showed a thickened stomach wall and enlarged lymph nodes, suggesting stomach cancer. She then had an FDG PET/CT scan, which showed moderate FDG uptake in the stomach wall, with a SUVmax of 2.9, and mild FDG uptake in the lymph nodes, with a SUVmax of 2.0. She also had a FAPI PET/CT scan, which showed mild uptake in the stomach lesion, with a SUVmax of 5.0, and less uptake in the lymph nodes, with a SUVmax of 1.4. An endoscopy revealed a stomach ulcer, and a biopsy confirmed stage II1 diffuse large B-cell lymphoma, a primary gastric lymphoma, rather than stomach cancer (26). In another case by Lu et al. (27) with swollen lymph nodes in his neck, armpits, and groin underwent biopsies, which confirmed diffuse large B-cell lymphoma (DLBCL). A subsequent FDG PET/CT scan showed high tracer uptake in the enlarged lymph nodes and significantly enlarged kidneys, suggesting lymphoma involvement in the kidneys (SUVmax: 18.98). However, a FAPI PET/CT scan performed three days later revealed a stark contrast: the lymph nodes and kidneys, which had shown high FDG uptake, showed minimal FAPI uptake. The kidneys did show diffuse FAPI uptake (SUVmax: 9.42), but this was interpreted as normal physiological activity, not disease. These findings highlight the notable differences between FDG and FAPI PET/CT in assessing DLBCL. Another case showed high [^68^Ga]FAPI uptake in primary thyroid DLBC possibly due to increased fibrosis from both the lymphoma and the patient's pre-existing thyroiditis. In this case, a 60-year-old woman, previously diagnosed with Hashimoto's thyroiditis presented with worsening neck swelling, breathing difficulties and a large, painless neck mass. Ultrasound showed diffuse thyroid enlargement and abnormal thyroid antibody levels were noted (TSH: 9.36 mIU/ml). Suspicion of a malignant thyroid tumor led to a [^68^Ga]FAPI PET/CT scan. The scan showed abnormal radiotracer uptake (SUVmax of 8.6) in the enlarged thyroid and a retrosternal goiter. A biopsy was performed, confirming diffuse large B-cell lymphoma (DLBCL) (28). In another case, a CT scan revealed a 2.5 cm mass in the anterior mediastinum of a 32-year-old woman, experiencing five months of breathing difficulties and occasional chest pain. A [^68^Ga]FAPI-04 PET/CT scan showed significant FAPI uptake in the mediastinal mass (SUVmax 9.7), and also in the pericardial fluid. Furthermore, increased FAPI uptake was noted in a paraesophageal lymph node (SUVmax 13.0), suggesting an invasive thymoma with lymph node metastasis. However, a CT-guided biopsy identified DLBCL (29). In further case, a 67-year-old woman experiencing left-sided weakness, facial paralysis, and walking difficulties had a brain MRI showing a 2.7 × 2.4 cm mass in her right frontal lobe. This mass, which displayed central necrosis, swelling, and increased enhancement, raised suspicion for lymphoma, glioma, or metastatic cancer. To further investigate, she underwent both FDG and FAPI PET/CT scans. The FDG scan showed high metabolic activity in the mass (SUVmax 9.6), while the FAPI scan showed only mild and uneven tracer uptake (SUVmax 3.1). With no other abnormalities found, the mass was surgically removed and identified as primary central nervous system diffuse large B-cell lymphoma. The lower FAPI uptake was likely due to the presence of fibrosis within the lymphoma (30).

## 4 Discussion

The role of [^18^F]FDG PET/CT in lymphoma has been widely established (5, 6). The role of [^18^F]FAPI PET/CT in the diagnostic and staging evaluation of lymphoma, remains relatively understudied compared to its application in other malignancies. Cancer-associated fibroblasts (CAFs) can either help or hinder tumor growth, depending on their specific characteristics. Recent research, such as the prospective study by Jin et al., has suggested that DLBCL exhibits moderate to strong fibroblast activation protein (FAP) expression, with all 34 patients in their cohort demonstrating positive lesions on FAPI PET. However, selected case reports present a compelling counterexample, highlighting the potential for complete absence of FAPI uptake in aggressive DLBCL. This observation suggests that [^18^F]FAPI PET/CT may not be a consistently reliable tool for staging DLBCL, where [^18^F]FDG PET/CT remains the gold standard. Given the established association between the stroma-1 gene signature and improved survival outcomes in DLBCL, Lu et al. (27) postulate that the observed low FAPI uptake in their case may reflect a distinct subgroup of patients with poorer prognosis. The lack of FAPI uptake could potentially indicate a less reactive stromal environment, which may correlate with a more aggressive tumor phenotype. Therefore, the authors propose that [^18^F]FAPI PET/CT, while potentially limited in staging DLBCL, may serve as a “reverse prognostic indicator.” Specifically, low or absent FAPI uptake could identify patients at higher risk of adverse outcomes, allowing for tailored therapeutic strategies and closer monitoring.

Beyond its potential utility in characterizing lymphoma subtypes, FAPI imaging could be particularly useful in specific anatomical locations, such as the brain, liver, and oropharynx, where it may enhance lymphoma diagnostic workup. Indeed, especially in non-FDG-avid subtypes, such as primary hepatic extranodal marginal zone MALToma, [^68^Ga]FAPI PET/CT has proved useful in the assessment of liver involvement (21).

Potential pitfall, particularly in mediastinal masses, may include thymoma or thymic carcinomas. It is important to bear in mind that FAPI may present high uptake in such tumors (18, 29, 31). Notably, FAPI SUVmax appears to be consistently higher in thymic carcinomas compared to thymomas (31).

While CD19 CAR-T cell therapy has greatly improved treatment for blood cancers, its use in solid tumors is challenging. This is because many cancer targets are also found in healthy tissues, potentially causing side effects. Additionally, tumor cells vary in their target expressions, and the tumor's surroundings can hide these targets, reducing CAR-T cell effectiveness. Therefore, a different approach targets non-cancerous cells that influence the tumor's environment. Cancer-associated fibroblasts are a promising target, leading to the development of FAP-targeted CAR-T cells. FAPI PET could be used to measure FAP levels and guide this therapy. For example, studies in mice with lung cancer showed that FAPI PET can track the response to FAP CAR-T cell therapy. It is anticipated that FAPI PET's use in relapsed or refractory lymphomas will be explored further (6).

In addressing the limitations of our review, it is essential to highlight that the available evidence remains preliminary. The data consists of only three small cohort studies and a dozen case reports, affecting the generalizability of our findings. Collectively, the evidence indicates that FAPI PET/CT may serve as a complement to [^18^F]FDG PET/CT, particularly in cases with low FDG avidity or challenging anatomical sites. However, its sensitivity is lower in lymphomas with minimal stromal FAP expression. Notably, variability imaging protocols and study designs highlights the pressing need for standardized acquisition and reporting.

Further research is warranted to validate the potential of FAPI PET/CT as a diagnostic tool and prognostic biomarker in lymphoma, especially in DLBCL. Large-scale prospective studies are needed to investigate the correlation between FAPI uptake, stromal characteristics, and clinical outcomes in lymphoma patients.

## 5 Conclusions

In conclusion, while FAPI PET/CT exhibits lower diagnostic sensitivity than [^18^F]FDG PET/CT in lymphomas characterized by low FAP expression, it retains potential as a complementary imaging modality. [^18^F]FDG PET/CT remains the gold standard of lymphoma imaging, but FAPI PET/CT may provide supplementary information regarding the molecular characteristics of lymphomas. While its overall role in routine staging appears less significant than that of [^18^F]FDG PET/CT, its prognostic and therapeutic implications warrant further research.

## Data Availability

The original contributions presented in the study are included in the article/Supplementary Material, further inquiries can be directed to the corresponding author.
