# Supplementary material for: The role of FAPI PET/CT in patients with lymphoma: a systematic review
Source: Front Nucl Med. 2025 May 9;5:1589903. doi: 10.3389/fnume.2025.1589903 (PMC12101064; doi:10.3389/fnume.2025.1589903)
Supplement: Supplementary file 1 [file Table1.docx]

| **Study** | **RISK OF BIAS** | | | | **APPLICABILITY CONCERNS** | | |
| --- | --- | --- | --- | --- | --- | --- | --- |
|  | **PATIENT SELECTION** | **INDEX TEST** | **REFERENCE STANDARD** | **FLOW AND TIMING** | **PATIENT SELECTION** | **INDEX TEST** | **REFERENCE STANDARD** |
| Chen et al. | ☺ | ☺ | ☺ | ☺ | ☺ | ☺ | ☺ |
| Jim et al. | ☺ | ☺ | ☺ | ☺ | ☺ | ☺ | ☺ |
| Hirmas et al. | ☹ | ☺ | ☺ | ☺ | ☹ | ☺ | ☺ |

☺Low Risk ☹High Risk ? Unclear Risk

**Table S2** Tabular presentation for QUADAS-2 results including all the original articles included in the systematic review
